# The effect of an information and communication technology (ICT) on older adults’ quality of life: study protocol for a randomized control trial

**DOI:** 10.1186/s13063-015-0713-2

**Published:** 2015-04-25

**Authors:** David H Gustafson, Fiona McTavish, David H Gustafson, Jane E Mahoney, Roberta A Johnson, John D Lee, Andrew Quanbeck, Amy K Atwood, Andrew Isham, Raj Veeramani, Lindy Clemson, Dhavan Shah

**Affiliations:** Center for Health Enhancement Systems Studies, University of Wisconsin-Madison, Madison, WI 53706 USA; Division of Geriatrics, Department of Medicine, University of Wisconsin School of Medicine and Public Health and Executive Director, Wisconsin Institute for Health Aging, Madison, WI 53792 USA; Department of Industrial and Systems Engineering, University of Wisconsin-Madison, Madison, WI 53705 USA; College of Engineering and School of Business and Executive Director, University of Wisconsin E-Business Institute, University of Wisconsin-Madison, Madison, WI 53706 USA; Aging, Work & Health Research Group, Faculty of Health Sciences, University of Sydney, Sydney, Australia; Mass Communication Research Center, School of Journalism and Mass Communication, University of Wisconsin-Madison, Madison, WI 53706 USA

**Keywords:** Older adults, Technology, eHealth, Gerontology

## Abstract

**Background:**

This study investigates the use of an information and communication technology (Elder Tree) designed for older adults and their informal caregivers to improve older adult quality of life and address challenges older adults face in maintaining their independence (for example, loneliness and isolation, falling, managing medications, driving and transportation).

**Methods/Design:**

This study, an unblinded randomized controlled trial, will evaluate the effectiveness and cost of Elder Tree. Older adults who are at risk for losing their independence - along with their informal caregivers, if they name them - are randomized to two groups. The intervention group has access to their usual sources of information and communication as well as to Elder Tree for 18 months while the control group uses only their usual sources of information and communication. The primary outcome of the study is older adult quality of life. Secondary outcomes are cost per Quality-Adjusted Life Year and the impact of the technology on independence, loneliness, falls, medication management, driving and transportation, and caregiver appraisal and mastery. We will also examine the mediating effect of self-determination theory. We will evaluate the effectiveness of Elder Tree by comparing intervention- and control-group participants at baseline and months 6, 12, and 18. We will use mixed-effect models to evaluate the primary and secondary outcomes, where pretest score functions as a covariate, treatment condition is a between-subjects factor, and the multivariate outcome reflects scores for a given assessment at the three time points. Separate analyses will be conducted for each outcome. Cost per Quality-Adjusted Life Year will be compared between the intervention and control groups. Additional analyses will examine the mediating effect of self-determination theory on each outcome.

**Discussion:**

Elder Tree is a multifaceted intervention, making it a challenge to assess which services or combinations of services account for outcomes in which subsets of older adults. If Elder Tree can improve quality of life and reduce healthcare costs among older adults, it could suggest a promising way to ease the burden that advancing age can place on older adults, their families, and the healthcare system.

**Trial registration:**

ClinicalTrials.govNCT02128789. Registered on 26 March 2014.

**Electronic supplementary material:**

The online version of this article (doi:10.1186/s13063-015-0713-2) contains supplementary material, which is available to authorized users.

## Background

Almost 90% of adults over 65 want to live in their homes as long as possible, also referred to as aging in place [1]. Challenges to aging in place include isolation and loneliness [2], falling [3], managing medications [4], and driving and transportation [5]. The cost of falls alone among US adults 65 and older has been estimated at $23.3 billion (in 2008 dollars) annually [6]. Nearly half (46.5%) of adults 65 and older have more than one chronic condition [7], and care for people with multiple chronic conditions accounts for an estimated 95% of Medicare spending [8]. Without innovative interventions, these and other costs are expected to escalate as the proportion of older adults in the population increases. Between 2005 and 2030, the number of US adults aged 65 and older will almost double, from 37 million to more than 70 million [9].

Technology may improve outcomes for older adults [10]. For instance, an eHealth program was effective in promoting health education among frail older people [11], and using the Internet for communication was associated with reduced loneliness [12]. However. few technological systems have been designed specifically for older users [13,14] or rigorously tested for effectiveness [15].

The goal of this study is to test the effects of a technology called Elder Tree designed for and used by older adults and their family caregivers. The primary purpose of Elder Tree, a web-based information and communication technology (ICT), is to improve older adult quality of life.

## Methods/Design

### Study design and hypotheses

The study is a randomized longitudinal trial conducted by the Active Aging Resource Center (AARC). AARC is a consortium of university, state, and community partners headquartered at the Center for Health Enhancement Systems Studies at the University of Wisconsin- Madison, WI, USA (CHESS). CHESS develops ICTs to help patients and their families improve their health and well-being. Previous CHESS ICTs have been proven effective in numerous randomized trials for a variety of conditions, including alcohol use disorders [16], lung cancer [17,18], pediatric asthma [19], breast cancer [20], and HIV [21]. CHESS also develops process improvement strategies for healthcare systems.

Participants in the study - older adults and their informal caregivers - are randomized to an intervention group that uses their usual sources of information and communication and has access to Elder Tree from laptops and other devices, or to a control group in which participants use only their usual sources of information and communication. Participants in the Elder Tree group receive access to Elder Tree for 18 months and, if needed, a computer and Internet service. The computer is almost always a touchscreen laptop; two participants have been given large monitors because of vision problems. Participants who have their own desktop, laptop, tablet, or smartphone use their own devices. Participants’ use of other services and interventions is not controlled during the trial in either group.

The primary hypothesis is that older adults assigned to Elder Tree will, compared with a control group, have improved quality of life (QOL). Secondary hypotheses are that older adults assigned to Elder Tree will have, compared with those in the control group, improved independence, lower healthcare costs per Quality-Adjusted Life Year (QALY), less loneliness, fewer falls, improved medication management, and greater ease of transportation and driving, and that informal caregivers with access to Elder Tree will have improved caregiver appraisal (reduced burden, increased mastery of caregiving, and increased satisfaction with the older adult receiving care), and improved coping strategies compared with caregivers without Elder Tree. We also hypothesize that self-determination theory (SDT) will mediate Elder Tree effects.

In addition to estimating healthcare costs in both groups, we will estimate the cost of delivering Elder Tree to determine what a governmental entity or other organization would pay to provide Elder Tree on a per-household basis.

### Intervention

Elder Tree builds upon ICTs created previously at CHESS for a variety of serious and chronic illnesses (for example, asthma, breast and lung cancer, addiction, and so on) and subjected to many randomized trials [16,18,19,22,23] and field tests [24,25]. Elder Tree has been developed by content experts collaborating with older adults, caregivers, and community and state partners, such as local Aging and Disability Resource Centers and the Wisconsin Bureau of Aging. Information about falls prevention was adapted from the Stepping On falls prevention program, with permission of its authors [26]. See Table 1 for a list of services in Elder Tree.

**Table 1:** **Elder Tree services**

**Purpose of service for user**	**Challenge being addressed**				
	**Isolation and loneliness**	**Driving and transportation**	**Caregiving**	**Medication management**	**Falls prevention**
Learn	Creating and sharing tips				
	Links to resources				
	Training to use the site (video tutorials, group training, paper manual)				
		Tips from driving coach	Tips from caregiver coach	Tips from diabetes diet coach	Tips from falls coach
					Videos for exercise and falls prevention
Communicate	Discussion groups, photo-sharing				
	Family and Friends (a discussion group for only a participant’s invited family and friends. Family and friends do not have access to the rest of Elder Tree)				
	Private messages				
	Ask a Coach: Elder Tree has four: a driving coach, caregiver support coach, diabetes diet coach, and falls prevention coach				
	Bulletin Board (a place where users post recipes, announcements of local events, and other information)				
Self-assess	During setup: personal assets and needs				
	For ongoing use: My Health Tracker and My Services. ‘My Health Tracker’ has 18 health measures (weight, blood pressure, quality of sleep, falls, and so on). Users choose which (if any) to track and how frequently they will respond to questions about the measures they select. The user sees results over time reported in graphic displays. In ‘My Services’, participants enter in-home services they receive. Then they get reminders about upcoming services and satisfaction surveys to complete, results of which users can track over time				
Use tools	Links to games				
	To-do list with reminders				
	Challenges from coaches to try healthy behaviors (for example, strength exercises)				
		Route planner			

The design of the Elder Tree interface and the services available in the system have been developed by working closely with hundreds of older adults. We learned from older adults the importance of enabling them to help one another (and not just receive help), keeping the technology safe from scams, having a simple interface that does not require computer savvy, and helping participants find community resources. From the many focus groups, interviews, and other interactions we had and continue to have with older adults, we have also learned how pervasive isolation and loneliness are. (Perhaps not surprisingly, the discussion groups have been the most heavily used service to date in the randomized trial of Elder Tree).

Members of the research team visit participants in their homes to set up and train them in Elder Tree. Researchers help each new participant - older adult and caregiver, if the older adult has named a caregiver - create an Elder Tree profile, which includes such information as interests and activities, ZIP code, and chronic conditions. Some of the information is used to tailor Elder Tree services to each user. For example, a user who is diabetic will receive tips and information from the diabetes diet coach and be invited to take part in a discussion group for participants who have diabetes. Use of Elder Tree is monitored. If an older adult or caregiver changes his or her pattern of using the system or stops using the system entirely after having participated, members of the research team call to find out if any system problems or other solvable issues are preventing their participation. The county coordinator -a grant-funded member of the research team who works in each of the 3 areas where the study is being conducted - calls a participant 1 week after the in-home training to answer questions or solve problems that may have arisen in using the system. Guidelines for the appropriate use of the technology are posted within Elder Tree. Participants whose use is inappropriate (for example, abusive to other participants in online discussion groups) are warned and, if necessary, discontinued. To date (11 February 2015), no participant has been discontinued.

### Theoretical foundation

Elder Tree rests on the foundation of SDT, like previous CHESS ICTs [27]. SDT holds that three basic psychological needs must be satisfied to foster well-being: autonomy, competence, and relatedness [28]. Elder Tree services have been designed to: (1) provide older adults with a sense of control over their situation (enhancing autonomy), (2) empower older adults and caregivers through information and skills training (enhancing competence), and (3) increase social support through links to other older adults and to experts (enhancing relatedness). Many services within Elder Tree relate to multiple SDT constructs (that is, acquiring information about preventing falls may improve both a participant’s competence and autonomy). According to SDT, healthcare contexts that support patients’ psychological needs for autonomy, competence, and relatedness will improve older adult QOL (older adults will have better physical and mental health) [29].

In evaluating past ICTs, we have also explored a more specific idea, connected to the relatedness construct in SDT, to explain a system’s effects. Receiving and, most importantly, expressing emotional content in online discussion groups have been shown to be associated with better outcomes [30-33]. We will also examine this outcome among Elder Tree participants.

### Ethics

The study received approval from the education and social/behavioral science institutional review board at the University of Wisconsin-Madison (reference number 2013–0171) and is registered at Clinical Trials.gov (NCT02128789). The study complies with the relevant Standard Protocol Items: Recommendations for Interventional Trials (SPIRIT) statement and World Health Organization (WHO) checklist (see Additional file 1). The study is funded by the United States Department of Health and Human Services Agency for Healthcare Research and Quality.

### Participants

Participants in the randomized trial are adults age 65 and older and their informal caregivers (spouses, children, or others who provide physical, emotional, and/or financial support for the older adult). Participants are being recruited from three regions in Wisconsin: urban Milwaukee County, suburban Waukesha County, and rural Richland, Juneau, and Sauk Counties. See Table 2 for inclusion and exclusion criteria. Inclusion criteria for older adults were selected because they represent risk factors for nursing home admission [34-37].

**Table 2:** **Inclusion and exclusion criteria for randomized clinical trial**

**Older adults**	
Inclusion	• Age 65 or older
criteria	• Live in one of three Wisconsin regions: Milwaukee County; Waukesha County; or Richland, Juneau, or Sauk Counties
	• In the last 12 months, has experienced one or more of the following:
	○ Fallen once or more
	○ Felt sad or depressed
	○ Received home-health services
	○ Stayed in a skilled nursing facility
	○ Gone to the emergency room
	○ Been admitted to the hospital
Exclusion criteria	• Is currently homeless or living in a hospice center, assisted living facility without access to a stove, or nursing home
	• Needs help getting into or out of a bed or a chair
**Informal caregivers**	
Inclusion criteria	• Age 18 or older
	• Provide physical, emotional, and/or financial support to the older adult
	• Be named by the older adult as a caregiver
Exclusion criteria	• Being cognitively impaired, as determined by the recruiter’s observation (for example, inability to answer questions or track the conversation)
**Both older adults and informal caregivers**	
Inclusion criteria	• Understand the consent form, which is in English
Exclusion criteria	• Being unable to give informed consent
	• Being unable to use Elder Tree (for example, poor vision that prevents reading a computer screen)

We changed our recruitment criteria from our original plans based on results of a pilot test. Originally, we planned to recruit older adults who had had, in the last 12 months, three of the bulleted inclusion criteria shown in Table 2, but these criteria proved to be too restrictive (that is, to eliminate potential participants who would likely benefit from Elder Tree) and, according to the county coordinators who did the screening, so time-consuming to evaluate and invasive of subjects’ privacy that they drove away potential participants. We have settled on recruiting patients who have one or more of the inclusion criteria.

### Recruitment

Each of the three regions in the study is served by an Aging and Disability Resource Center (ADRC). ADRCs are state funded, single- or multi-county agencies in Wisconsin that connect older people and people with disabilities to information, assistance, and counseling. A county coordinator works out of each local ADRC office to recruit potential participants for the study. The county coordinators raise awareness and interest among older adults by giving presentations about the study in neighborhood centers, churches, clubs, congregate eating facilities, and other places. The county coordinators explain the study and the eligibility criteria and ask attendees to provide their contact information. To encourage older adults to provide their names, the coordinators offer a drawing at the end of a presentation in which one of the slips of paper with contact information will be drawn to receive a $10 gift certificate to a local restaurant. Older adults and caregivers who contact an ADRC are also encouraged to participate.

**Figure 1:**
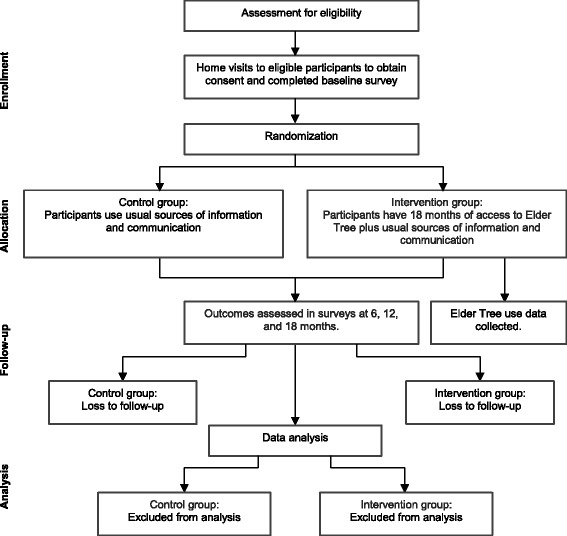
Participant flow.

Older adults who provide their contact information are called to confirm eligibility. If the older adult is eligible (and the informal caregiver, if the older adult names an informal caregiver who is also eligible), the county coordinator sends the baseline survey in the mail and sets up a time for a home visit. During the home visit, the county coordinator explains consent and obtains the completed consent form (see the forms in Additional files 2 and 3) and baseline survey from the older adult (and caregiver, if there is one). To date (31 March 2015), we have recruited 392 older adult participants to the study. We plan to recruit 450 participants. Figure 1 shows the flow of participants through the trial.

### Randomization

The project statistician used a computer-generated allocation sequence to randomize eligible older adults (or older adult/caregiver dyads) in a 1:1 ratio to intervention (Elder Tree) or control. Randomization is stratified by geographic region, whether a participant has his or her own computer or other device, and living alone, using random blocks of 4 and 6. Randomization is implemented by the project director using sequentially numbered, sealed, opaque envelopes. The sequence is thus unknown to the county coordinators who enroll participants. Because of the nature of the intervention, neither participants nor research staff are blinded to allocation.

### Measures and data collection

Our assessment battery consists of instruments with established reliability and validity. Survey measures were chosen based on our theoretical model, SDT. We also sought measures with an easy reading level. Surveys are identified by a code, not a name, and mailed to participants. The form linking codes and names is kept in REDCap (Research Electronic Data Capture) [38]. REDCap is a secure, web-based application designed to support data management for research. Data are stored in REDCap; data management procedures are described the code book. Table 3 summarizes the variables and measures we are using, their evaluation schedule, and item-response burden.

**Table 3:** **Outcome and other measures**

**Outcome measures**									
**Construct/Associated Hypothesis** ^**a**^		**Instrument**	**Burden**	**When**					**Who**
				**S**	**B**	**6**	**12**	**18**	
Primary outcome:									
Quality of life: Global Mental Health and Global Physical Health/1		PROMIS Global Health [39]	10		X	X	X	X	OA
									CG
Secondary outcomes:									
Independence: Activities of Daily Living (ADLs) and Instrumental Activities of Daily Living (IADLs)/2		Getting to places outside the home, moving/walking around the home, taking your medications, planning and preparing meals, bathing and using the toilet, dealing with finances [53-55]	6		X	X	X	X	OA
Cost per Quality-Adjusted		QALY: PROMIS Global Health converted to QALYs using EuroQol-5D (EQ-5D) [40]	0		X	X	X	X	OA
									CG
Life Year (QALY)/3									
		Intervention cost: modified DATCAP [41]	0			X		X	OA
									CG
		Healthcare utilization: patient survey using modified medical services utilization form [43]	6		X	X	X	X	OA
									CG
Loneliness/4		UCLA Loneliness Scale (Version 3) [56]	20		X	X	X	X	OA
									CG
Falls/5		Recent falls: # of falls; # requiring medical attention	2		X	X	X	X	OA
		Falls risk: Falls Behavioral Scale for the Older Person (FaB) (modified) [57]	15		X	X	X	X	
Medication management/6		Presence of risky medication: status of taking blood thinners, insulin, or oral medications for high blood sugar or diabetes [58]	3		X	X	X	X	OA
		Medication adherence: to what extent would you estimate that you take your medication doses?	1		X	X	X	X	
		Medication side effects: presence or absence of common side effects of antiplatelets/anticoagulants and insulin/oral hypoglycemics [59]	18		X	X	X	X	
Ease of driving and transportation/7		Ease of/comfort with transportation; # of crashes and near-misses	7 to 12		X	X	X	X	OA
Caregiver appraisal, burden, satisfaction with relationship, and mastery/8		Lawton Caregiving Appraisal Scale [60]	19		X	X	X	X	CG
Caregiver coping: wishfulness, acceptance, intra-psychic, instrumental/9		Caregiver Coping Strategies [61]	16		X	X	X	X	CG
Mediators: three constructs of self-determination theory		Autonomy: *Survey:* How well do you carry out social activities and roles; do you currently drive? *Elder Tree use data:* # and extent of services used.	2		X	X	X	X	OA
									CG
		Competence: *Survey:* To what extent can you carry out everyday activities; IADLs, comfort with technologies)? *Elder Tree use data:* # of informational pages and tips viewed.	3		X	X	X	X	OA
									CG
		Relatedness: *Survey:* selected items from the MOS Social Support Survey (MOS-SS) (modified) [62] related to how often someone is there to *provide* support to and *receive* support from you, plus how often you have participated in a health or medical support group or social club or group? *Elder Tree use data:* # of emotionally supportive messages posted and read	17		X	X	X	X	OA
									CG
**Other measures**									
**Construct**		**Instrument**	**Burden**	**When**					**Who**
				**S**	**B**	**6**	**12**	**18**	
Depression		Patient Health Questionnaire (PHQ-8) [63]	8		X	X	X	X	OA
									CG
Living arrangement		Setting (for example, own home/apartment); live alone or not [34]	2	X	X	X	X	X	OA
Elder Tree system use		Server log files: pages, minutes, services, engagement, patterns, comments, and so on	0		Constantly while participants use Elder Tree.				OA
									CG
Comfort with technology		Smartphone/tablet; desktop/laptop computer; Email; Facebook	4		X	X	X	X	OA
									CG
Physical limitations to technology use		Auditory, visual, motor, other	1		X	X	X	X	OA
**Measures related to other challenges**									
**Challenge**	**Construct**	**Instrument**	**Burden**	**When**					**Who**
				**S**	**B**	**6**	**12**	**18**	
Social con-nectedness	Online bonding	CHESS Bonding Scale	4		X	X	X	X	OA
									CG
	Size of social network	# of people to listen to you, to whom you listen, from whom you get help, show love, get together to do something enjoyable	5		X	X	X	X	OA
	Other support	Types of therapy or support groups	6		X	X	X	X	OA
									CG
Service delivery	Satisfaction with service delivery	Showering/bathing/grooming; in-home meal prep or Meals on Wheels; toileting and incontinence; medical support services	4		X	X	X	X	OA
									CG

^a^Hypotheses: compared with the control group, at 6, 12, and 18 months, those randomized to Elder Tree will experience:

1. Improved quality of life (older adults).

2. Improved independence (older adults).

3. Lower healthcare cost per QALY (older adults).

4. Less loneliness (older adults).

5. Fewer falls (older adults).

6. Improved medication management (older adults).

7. Greater ease of transportation and driving (older adults).

8. Improved caregiver appraisal (caregivers).

9. Increased coping strategies (caregivers).

Mediation hypothesis: SDT (autonomy, competence, relatedness) will mediate the effects of Elder Tree on older adult outcomes.

S, screening; B, baseline; OA, older adult; CG, caregiver.

CHESS, Center for Health Enhancement Systems Studies at the University of Wisconsin-Madison, WI, USA; DATCAP, Drug Abuse Treatment Cost Analysis Program; MOS; Medical Outcomes Study; PROMIS, Patient-Reported Outcomes Measurement Information System.

We are also collecting data on the server about how participants use Elder Tree. We use this information to monitor the functioning of the website - for example, see if the website has problems. These use data also will be analyzed after the randomized trial to explore such questions as whether participants who used Elder Tree the most had better outcomes.

#### Measures related to QOL

The primary outcome of older adult quality of life is measured using the Patient-Reported Outcomes Measurement Information System (PROMIS) Global Health scale, a 10-item subjective measure of general health [39]. It includes a 4-item global physical health scale (Cronbach’s α = 0.81), a 4-item global mental health scale (Cronbach’s α = 0.86), and 2 additional items - general health and satisfaction with social roles - that can each be scored as a single item. PROMIS scales were developed using item response theory and capture a greater range of the trait being measured, with greater precision, than other instruments.

#### Measures related to costs

To quantify the tradeoff between the hypothesized improvements in QOL that Elder Tree may produce versus the cost of providing Elder Tree, we will use incremental cost-effectiveness ratios (ICERs). An ICER is the ratio of the change in costs of an intervention (compared with an alternative) to the change in the effect(s) of the intervention. The primary ICER will be incremental cost per increased QALY. QALYs will be calculated using the approach outlined by Revicki, *et al*. [40].

We will collect data for two types of costs: intervention cost and healthcare utilization cost. Intervention cost will be estimated using a modified version of The Drug Abuse Treatment Cost Analysis Program (DATCAP) [41]. The DATCAP instrument was originally developed for addiction treatment programs [41]. It has been previously adapted to assess the cost of a quality improvement intervention for addiction treatment [42] and has been adapted for use in this study. We will track: (1) system development costs, including costs associated with programming Elder Tree; (2) research costs, which include salaries and fringe benefits for members of the research team to collect outcome measures; (3) set-up and implementation costs, including salaries (with fringe benefits) for county coordinators; and (4) operating costs, such as hardware (touchscreen laptops), monthly data plans, information technology (IT) costs for maintaining the system, and research staff time for monitoring system use. We will make set-up, implementation, and operating costs available because we anticipate that potential payers (governmental agencies, insurance companies, health systems) will be most interested in these costs.

Healthcare utilization will be collected via bi-annual patient surveys using a modified version of the medical services utilization form [43]. Participants will be asked about their visits to the emergency room and urgent care, hospitalizations, and use of assisted-living facilities and nursing homes. A 6-month recall period is used. Per-patient estimates use average cost per day/episode from the literature multiplied by self-reported healthcare use collected via the medical services utilization form [44-47].

Summing the costs of the intervention and healthcare utilization provides an estimate of total costs for each participant. Costs and effects will be measured at the individual patient/household level, which will allow us to generate cost-effectiveness acceptability curves to represent uncertainty in the ICERs between the intervention and control groups [48].

#### Measures related to caregivers and mediation

Two instruments will assess the results of using Elder Tree on caregivers. A combination of survey items and Elder Tree use data will assess the mediating effect of the three constructs of SDT. Computer-aided content analyses of discussion group posts will be used to explore the relationship between the reception and expression of emotional content and outcomes.

### Sample size

We originally targeted a sample size of N = 300 (150 per group), which would provide 80% power to detect an effect size of Cohen’s d = .4, assuming a response rate near 80% (similar to that of other CHESS studies [16]). Given our diffuse system, we made a decision to extend our focus to individuals and dyads that would benefit from one or two aspects of our system rather than multiple features, with a larger sample therefore needed to detect the greater range of incremental effects on discrete outcomes. More specifically, we set a revised target sample size of N = 450 (225 per group), which would provide 80% power to detect a smaller effect size of Cohen’s d = .3 with an 80% response rate.

### Timeline

**Figure 2:**
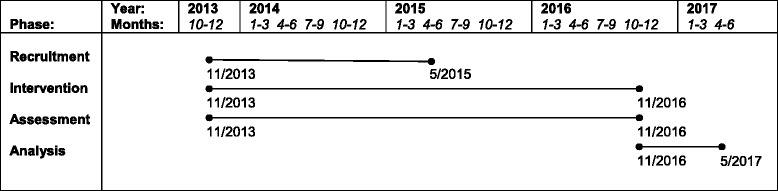
Timeline.

Recruitment began in November 2013 and will end in May 2015. The intervention period will end in November 2016. Figure 2 shows the timeline and status of the trial.

### Data analysis

We will evaluate the effects of Elder Tree by comparing intervention and control participants at 6-, 12-, and 18-month assessments. We will use mixed-effects models to evaluate our primary and secondary hypotheses, where pretest score functions as a covariate, treatment condition is a between-subjects factor, and the multivariate outcome reflects scores for a given assessment across the 3 time points (months 6, 12, and 18). Separate analyses will be conducted for each outcome.

We will use path analysis to test whether the constructs of SDT mediate the relationship between Elder Tree and participants’ QOL at 6, 12, and 18 months. Mediation will be tested based on the significance and size of the specific indirect effects of system use through SDT constructs on relevant outcomes [49-51], as well as the overall fit of the model. Overall model fit will be assessed with statistics traditionally used for this task (for example, CFI, TLI, RMSEA).

Secondary analyses will lend further insight related to primary hypotheses’ findings. The intercorrelations between studied outcomes (for example, Instrumental Activities of Daily Living (IADLs), QOL) and mediator (SDT) will clarify whether these outcomes represent unique versus highly-correlated outcomes. Additional analysis will disaggregate overall Elder Tree effects into component services. Given the non-random selection of services and the potential correlation between the use of certain services, this analysis will address whether a service was used and the intensity of that use. This should reduce multi-collinearity issues in regression analysis and provide insights into each service’s contributions. Outcomes associated with specific Elder Tree services will also be compared between users and non-users of specific services in an exploratory Complier Average Causal Effect (CACE) analysis to examine whether actual use of a service (rather than randomized access to it) is associated with improved outcomes. We will also examine trends within groups over time (for example, whether gains from Elder Tree at 6 and 12 months are maintained to 18 months).

Because we anticipate that Elder Tree will take at least 3 months to have an effect, we plan to conduct exploratory analyses that mirror the aforementioned analyses but exclude participants who die or move to a nursing home or assisted living facility within the first 3 months of the intervention.

### Qualitative data collection and analysis

We have used multiple methods of data collection to plan, develop, and implement Elder Tree, including interviews, surveys, and focus groups with older adults and informal caregivers and testing of the technology as it developed. During the planning phase, we collaborated with the ADRC in each of the three regions participating in the study to identify the assets and challenges of older adults by using a community-based strategy called Asset-Based Community Development [52]. We held meetings with ADRC staff and teams of citizens to develop a plan to interview residents and combine the results. In each area, the three most important challenges identified through the process were identical: isolation and loneliness, how to know about and take advantage of community activities and resources, and transportation. During development, we tested paper prototypes and onscreen iterations of the technology with 335 older adults and caregivers one-on-one and in small groups. During implementation, we continue to digitally collect use data to help us understand and improve the system: for example, use data allow us to see which services in Elder Tree are least used. Knowing this, we can find out why participants are not using a service and how we might improve it. Continuous improvement is a hallmark of our approach to technology as we strive to keep our systems abreast of new or improved operating systems, changing conventions, and other developments.

## Discussion

The development and testing of Elder Tree mark several important advances. Very few technologies have been designed for or rigorously tested with older adults, who often have physical and cognitive limitations not common among younger people. Elder Tree has been developed with the deep involvement of older adults. The technology also has been developed by working closely with community and state partners, such as local Aging and Disability Resource Centers and the Wisconsin Bureau of Aging, both to create a technology that is adaptable to different communities and to build a base for dissemination. Because we will be able to relate outcomes to use of the system, we also hope to understand why and how the system works or does not work and for whom.

Elder Tree is a multifaceted intervention with interacting services. For example, better medication management as a result of using Elder Tree may reduce the risk of falling. These interactions may make it challenging to assess which services or combination of services in Elder Tree account for changes in outcomes. Settling on our recruitment criteria has been an unexpected challenge because our original inclusion criteria proved to be too restrictive.

We have undertaken an active dissemination campaign in Wisconsin from which we hope to learn about effective ways to promote the use of technology among older adults, including those who have not used computers before. By piloting dissemination during the randomized trial phase, we are learning what communities will need to be able to adapt and maintain Elder Tree for their residents and what types of community organizations may want to provide it and why. This early dissemination work will accelerate the transition from research to practice should the randomized trial show positive results.

If Elder Tree can improve the quality of life of older adults and reduce healthcare costs, it could suggest one path to significantly reducing the physical, emotional, and financial burdens that advancing age can place on older adults, their families and communities, and the healthcare system.

## Trial status

The trial has received ethical approval and recruited 392 participants to date (31 March 2015). We anticipate ending recruitment in May 2015.

## Additional files

Additional file 1:
**Completed SPIRIT checklist, the addendum to which contains the complete WHO checklist: SPIRIT_Fillable-checklist-15-Aug-2013.doc.**
Additional file 2:
**Consent form for older adults: AARC_OA Consent Form Approved_2_14.pdf.**
Additional file 3:
**Consent form for informal caregivers: AARC_CG Consent Form Approved_2_14.pdf.**

## Abbreviations

AARC: Active Aging Resource Center
ADLs: Activities of Daily Living
ADRC: Aging and Disability Resource Center
CACE: Complier Average Causal Effect
CHESS: Center for Health Enhancement Systems Studies at the University of Wisconsin-Madison, WI, USA
DATCAP: Drug Abuse Treatment Cost Analysis Program
IADLs: Instrumental Activities of Daily Living
ICERs: Incremental cost-effectiveness ratios
ICT: Information and communication technology
IT: Information technology
PROMIS: Patient-Reported Outcomes Measurement Information System
QALY: Quality-Adjusted Life Year
QOL: Quality of life
REDCap: Research Electronic Data Capture
SDT: Self-determination theory
SPIRIT: Standard Protocol Items: Recommendations for Interventional Trials
WHO: World Health Organization
