# Supplementary material for: The effect of an information and communication technology (ICT) on older adults’ quality of life: study protocol for a randomized control trial
Source: Trials. 2015 Apr 25;16:191. doi: 10.1186/s13063-015-0713-2 (PMC4417513; doi:10.1186/s13063-015-0713-2)
Supplement: Additional file 1: — Completed SPIRIT checklist, the addendum to which contains the complete WHO checklist: SPIRIT_Fillable-checklist-15-Aug-2013.doc. [file 13063_2015_713_MOESM1_ESM.doc]

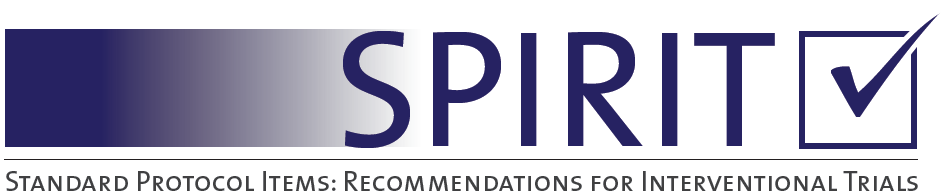


SPIRIT 2013 Checklist: Recommended items to address in a clinical trial protocol and related documents*

| Section/item | Item No | Description | Addressed on page number |
| --- | --- | --- | --- |
| **Administrative information** | | |  |
| Title | 1 | Descriptive title identifying the study design, population, interventions, and, if applicable, trial acronym | ______1______ |
| Trial registration | 2a | Trial identifier and registry name. If not yet registered, name of intended registry | ______3______ |
|  | 2b | All items from the World Health Organization Trial Registration Data Set | Addendum at the end of this document. |
| Protocol version | 3 | Date and version identifier This protocol is version 1.0, dated February 10, 2015. | __Here in yellow_ |
| Funding | 4 | Sources and types of financial, material, and other support | ______18_____ |
| Roles and responsibilities | 5a | Names, affiliations, and roles of protocol contributors | ____17-18_____ |
|  | 5b | Name and contact information for the trial sponsor Jonathan White: jonathan.white@ahrq.hhs.gov | __Here in yellow_ |
|  | 5c | Role of study sponsor and funders, if any, in study design; collection, management, analysis, and interpretation of data; writing of the report; and the decision to submit the report for publication, including whether they will have ultimate authority over any of these activities | ______18_____ |
|  | 5d | Composition, roles, and responsibilities of the coordinating centre, steering committee, endpoint adjudication committee, data management team, and other individuals or groups overseeing the trial, if applicable (see Item 21a for data monitoring committee)  David H. Gustafson Sr., PI, directs the Active Aging Research Center. The Center consists of a National Advisory Board (chair: Gustafson Sr), a steering committee (chair: Jane E. Mahoney), methods and data management core (chair: Dhavan Shah), a technical core (chair: Susan Dinauer), and a patient/consumer core (chair: David H. Gustafson Jr.). Day-to-day management of the study is done by Project Director McTavish. | __Here in yellow__ |
| Introduction |  |  |  |
| Background and rationale | 6a | Description of research question and justification for undertaking the trial, including summary of relevant studies (published and unpublished) examining benefits and harms for each intervention | ______4______ |
|  | 6b | Explanation for choice of comparators  We are comparing older adults’ (a) usual sources of information and communication to their (b) use of the usual sources plus Elder Tree because we want to understand whether Elder Tree causes an improvement in the status quo. | __Here in yellow_ |
| Objectives | 7 | Specific objectives or hypotheses | ____5-6______ |
| Trial design | 8 | Description of trial design including type of trial (eg, parallel group, crossover, factorial, single group), allocation ratio, and framework (eg, superiority, equivalence, noninferiority, exploratory) | ______5______ |
| Methods: Participants, interventions, and outcomes | | |  |
| Study setting | 9 | Description of study settings (eg, community clinic, academic hospital) and list of countries where data will be collected. Reference to where list of study sites can be obtained | ______9______ |
| Eligibility criteria | 10 | Inclusion and exclusion criteria for participants. If applicable, eligibility criteria for study centres and individuals who will perform the interventions (eg, surgeons, psychotherapists) | ______20_____ |
| Interventions | 11a | Interventions for each group with sufficient detail to allow replication, including how and when they will be administered | ___6-7; 19____ |
|  | 11b | Criteria for discontinuing or modifying allocated interventions for a given trial participant (eg, drug dose change in response to harms, participant request, or improving/worsening disease) | ______7_____ |
|  | 11c | Strategies to improve adherence to intervention protocols, and any procedures for monitoring adherence (eg, drug tablet return, laboratory tests) | ______7_____ |
|  | 11d | Relevant concomitant care and interventions that are permitted or prohibited during the trial | ______5_____ |
| Outcomes | 12 | Primary, secondary, and other outcomes, including the specific measurement variable (eg, systolic blood pressure), analysis metric (eg, change from baseline, final value, time to event), method of aggregation (eg, median, proportion), and time point for each outcome. Explanation of the clinical relevance of chosen efficacy and harm outcomes is strongly recommended | ____21-23____ |
| Participant timeline | 13 | Time schedule of enrolment, interventions (including any run-ins and washouts), assessments, and visits for participants. A schematic diagram is highly recommended (see Figure) | _14 + Figure 2__ |
| Sample size | 14 | Estimated number of participants needed to achieve study objectives and how it was determined, including clinical and statistical assumptions supporting any sample size calculations | _____13______ |
| Recruitment | 15 | Strategies for achieving adequate participant enrolment to reach target sample size | ____9-10_____ |
| **Methods: Assignment of interventions (for controlled trials)** | | |  |
| Allocation: |  |  |  |
| Sequence generation | 16a | Method of generating the allocation sequence (eg, computer-generated random numbers), and list of any factors for stratification. To reduce predictability of a random sequence, details of any planned restriction (eg, blocking) should be provided in a separate document that is unavailable to those who enrol participants or assign interventions | ____10______ |
| Allocation concealment mechanism | 16b | Mechanism of implementing the allocation sequence (eg, central telephone; sequentially numbered, opaque, sealed envelopes), describing any steps to conceal the sequence until interventions are assigned | _____10______ |
| Implementation | 16c | Who will generate the allocation sequence, who will enrol participants, and who will assign participants to interventions | _____10______ |
| Blinding (masking) | 17a | Who will be blinded after assignment to interventions (eg, trial participants, care providers, outcome assessors, data analysts), and how | _____10______ |
|  | 17b | If blinded, circumstances under which unblinding is permissible, and procedure for revealing a participant’s allocated intervention during the trial | _Does not apply__ |
| **Methods: Data collection, management, and analysis** | | |  |
| Data collection methods | 18a | Plans for assessment and collection of outcome, baseline, and other trial data, including any related processes to promote data quality (eg, duplicate measurements, training of assessors) and a description of study instruments (eg, questionnaires, laboratory tests) along with their reliability and validity, if known. Reference to where data collection forms can be found, if not in the protocol | _11-13 + Table 3_ |
|  | 18b | Plans to promote participant retention and complete follow-up, including list of any outcome data to be collected for participants who discontinue or deviate from intervention protocols  Participants who have not returned their 6-, 12-, and 18-month surveys will be called if they have not completed the survey in two weeks. No outcome data will be used after participants discontinue their involvement in the study. | _7 + here in yellow |
| Data management | 19 | Plans for data entry, coding, security, and storage, including any related processes to promote data quality (eg, double data entry; range checks for data values). Reference to where details of data management procedures can be found, if not in the protocol | _____11______ |
| Statistical methods | 20a | Statistical methods for analysing primary and secondary outcomes. Reference to where other details of the statistical analysis plan can be found, if not in the protocol | ____14-16_____ |
|  | 20b | Methods for any additional analyses (eg, subgroup and adjusted analyses) | ____14-15_____ |
|  | 20c | Definition of analysis population relating to protocol non-adherence (eg, as randomised analysis), and any statistical methods to handle missing data (eg, multiple imputation)  In previous work^1^, we completed 85% of 4-, 8-, and 12-month interviews. We anticipate completions reaching about 75% by month 18 in this study. We also kept missing data on core items within an interview to about 2% and expect to continue that rate in this study. Participants who do not complete interviews may have valid and non-ignorable reasons, e.g., some may not want to disclose information that may lead to a loss of governmental support. Multiple imputations will replace the small amounts of missing data to produce the least biased estimate for each analysis.^2^ Analyses will be rechecked by running them without missing data. If there are differences, we will use a general latent variable framework^3^ to analyse non-ignorable missingness that assumes that missing data are based in part on latent developmental trends and qualitatively different types of development by condition.   1. Gustafson DH, McTavish FM, Chih MY, Atwood AK, Johnson RA, Boyle MG, et al. A smartphone application to support recovery from alcoholism: a randomized clinical trial. JAMA Psychiatry 2014, 71: 566-572. 2. Schafer JL, Graham JW. Missing data: our view of the state of the art. Psychol Methods 2002, **7:** 147. 3. Muthen B, Asparouhov T, Hunter AM, Leuchter AF. Growth modeling with nonignorable dropout: alternative analyses of the STAR*D antidepressant trial. Psychol Methods 2011, **16:** 17-33. | __Here in yellow__ |
| **Methods: Monitoring** | | |  |
| Data monitoring | 21a | Composition of data monitoring committee (DMC); summary of its role and reporting structure; statement of whether it is independent from the sponsor and competing interests; and reference to where further details about its charter can be found, if not in the protocol. Alternatively, an explanation of why a DMC is not needed  Our DMC consists of three faculty members at the University of Wisconsin-Madison: Paul R Hutson (PharmD, MS, School of Pharmacy, Practice Division; committee chair), Daniel L. Mulkerin (MD, Dept. of Medicine, Section on Medical Oncology), and Jens C. Eickhoff (PhD, Dept. of Biostatistics and Medical Informatics). The DMC functions independently of the sponsor of the study, the PI and research team, and competing interests. Every year, we on the research team prepare a report about the progress of the study for the DMC and then meet with the DMC to discuss the state of the trial. After this process, we receive a letter approving of or raising further questions about our research. Further details about the DMC can be found at the Center for Health Enhancement Systems Studies network at the University of Wisconsin – Madison. | __Here in yellow__ |
|  | 21b | Description of any interim analyses and stopping guidelines, including who will have access to these interim results and make the final decision to terminate the trial  No interim analyses are being conducted on the primary and secondary outcomes. A decision to terminate the trial would be made by Jane Mahoney, MD, chair of the steering committee, and PI David Gustafson Sr. | __Here in yellow__ |
| Harms | 22 | Plans for collecting, assessing, reporting, and managing solicited and spontaneously reported adverse events and other unintended effects of trial interventions or trial conduct  In accordance with federal law and University of Wisconsin-Madison regulations, any human subjects issues that arise during the trial will be reported to the IRB. | __Here in yellow__ |
| Auditing | 23 | Frequency and procedures for auditing trial conduct, if any, and whether the process will be independent from investigators and the sponsor  As noted in the response to item 21a, the trial is subjected to an annual review by the independent Data Monitoring Committee. In addition, we have access to this committee throughout the year if the need arises. | __Here in yellow__ |
| Ethics and dissemination | | |  |
| Research ethics approval | 24 | Plans for seeking research ethics committee/institutional review board (REC/IRB) approval | ______8_______ |
| Protocol amendments | 25 | Plans for communicating important protocol modifications (eg, changes to eligibility criteria, outcomes, analyses) to relevant parties (eg, investigators, REC/IRBs, trial participants, trial registries, journals, regulators) Any changes will be submitted to and approved by the IRB. | __Here in yellow__ |
| Consent or assent | 26a | Who will obtain informed consent or assent from potential trial participants or authorised surrogates, and how (see Item 32) | ______10_____ |
|  | 26b | Additional consent provisions for collection and use of participant data and biological specimens in ancillary studies, if applicable | _Does not apply__ |
| Confidentiality | 27 | How personal information about potential and enrolled participants will be collected, shared, and maintained in order to protect confidentiality before, during, and after the trial | ______11_____ |
| Declaration of interests | 28 | Financial and other competing interests for principal investigators for the overall trial and each study site | _____17______ |
| Access to data | 29 | Statement of who will have access to the final trial dataset, and disclosure of contractual agreements that limit such access for investigators  The PI (Gustafson Sr.), steering committee chair (Mahoney), project director (McTavish), methods and data management core chair (Shah), and project statistician (Atwood) will have access to to the final dataset. No contractual agreements limit access for investigators. | __Here in yellow__ |
| Ancillary and post-trial care | 30 | Provisions, if any, for ancillary and post-trial care, and for compensation to those who suffer harm from trial participation None are planned. | __Here in yellow__ |
| Dissemination policy | 31a | Plans for investigators and sponsor to communicate trial results to participants, healthcare professionals, the public, and other relevant groups (eg, via publication, reporting in results databases, or other data sharing arrangements), including any publication restrictions  We plan to disseminate results of the trial through publication, without restrictions, regardless of the direction or magnitude of its effects. | __Here in yellow__ |
|  | 31b | Authorship eligibility guidelines and any intended use of professional writers | _____17 - 18____ |
|  | 31c | Plans, if any, for granting public access to the full protocol, participant-level dataset, and statistical code  Once we have completed our analysis, we will make the data available to researchers who contact us and provide evidence that their research has been approved by appropriate review bodies. | __Here in yellow__ |
| Appendices |  |  |  |
| Informed consent materials | 32 | Model consent form and other related documentation given to participants and authorised surrogates  The consent forms are submitted with the manuscript as separate files. | __Here in yellow__ |
| Biological specimens | 33 | Plans for collection, laboratory evaluation, and storage of biological specimens for genetic or molecular analysis in the current trial and for future use in ancillary studies, if applicable | _Does not apply__ |

*It is strongly recommended that this checklist be read in conjunction with the SPIRIT 2013 Explanation & Elaboration for important clarification on the items. Amendments to the protocol should be tracked and dated. The SPIRIT checklist is copyrighted by the SPIRIT Group under the Creative Commons “[Attribution-NonCommercial-NoDerivs 3.0 Unported](http://www.creativecommons.org/licenses/by-nc-nd/3.0/)” license.

**Addendum:**

**WHO Checklist**

Primary registry and trial identifying number: ClinicalTrials.govNCT02128789

Date of registration in primary registry: 26 March 2014

Secondary identifying numbers: HS019917, 5P50HS019917-04

Sources of monetary or material support: Agency for Healthcare Research and Quality; Epic Systems Corporation

Primary sponsor: Agency for Healthcare Research and Quality

Secondary sponsor: Epic Systems Corporation

Contact for public queries: DHG Sr, PhD, at 608-263-4882; [dhgustaf@wisc.edu](mailto:dhgustaf@wisc.edu)

Contact for scientific queries: DHG Sr, PhD, at 608-263-4882; [dhgustaf@wisc.edu](mailto:dhgustaf@wisc.edu)

Public title: Bringing Communities and Technology Together for Healthy Aging (Elder Tree)

Scientific title: same as above

Countries of recruitment: USA

Health condition(s) or problems(s) studied: challenges (such as loneliness, falls, managing medications, and transportation) that threaten the ability of older adults to live independently

Interventions: Intervention group uses usual sources of information and communication plus Elder Tree (a computer-based technology). Control group uses only usual sources of information and communication.

Key inclusion and exclusion criteria:

Ages eligible for study: ≥ 65 years

Sexes eligible for study: Both

Accepts eligible volunteers: Yes

Inclusion criteria for older adults: Live in Milwaukee, Waukesha, Richland, Juneau, or Sauk Counties, WI, USA; in the last 12 months, has experienced one or more of the following: fallen once or more, felt sad or depressed, received home-health services, stayed in a skilled nursing facility, gone to the emergency room, been admitted to the hospital

Exclusion criteria for older adults: Is currently homeless or living in a hospice center, assisted living facility without access to a stove, or nursing home

Study type: Interventional; allocation: randomized; unblinded; two arms, parallel; purpose: improve quality of life among older adults

Date of first enrolment: November 2013

Target sample size: 300 (150 per arm) after dropouts

Recruitment status: Recruiting

Primary outcome: Improved quality of life; time frame: 6, 12, and 18 months

Key secondary outcomes: Cost per Quality-Adjusted Life Year; impact of the technology on independence, loneliness, falls, medication management, driving and transportation, caregiver satisfaction and coping
