# Supplementary material for: The effect of an information and communication technology (ICT) on older adults’ quality of life: study protocol for a randomized control trial
Source: Trials. 2015 Apr 25;16:191. doi: 10.1186/s13063-015-0713-2 (PMC4417513; doi:10.1186/s13063-015-0713-2)
Supplement: Additional file 2: — Consent form for older adults: AARC_OA Consent Form Approved_2_14.pdf. [file 13063_2015_713_MOESM2_ESM.pdf]

AARC RCT Consent Form – for Older Adults

Study: Bringing Communities and Technologies Together for Healthy Aging - Randomized Control Trial
Participating Institution: University of Wisconsin- Madison (UW-Madison)
Investigators: David H. Gustafson, Ph.D., Active Aging Resource Center Principal Investigator

Invitation and Purpose: Staying safe at home is very important as we age. **You are being invited to take part in this study because you are 65 years of age or older and a Wisconsin resident living in Milwaukee, Richland, Sauk, Crawford, Juneau or Waukesha counties.** This study is being conducted by UW-Madison and with members of the State of Wisconsin Aging and Disability Resource Centers (ADRC). This study will help us learn how technology can support older adults and their families.

What will I have to do?

1. Participation in the study is completely voluntary and will last a total of 18 months. After you join the study, you and your caregiver, if you have one, will be randomly put in one of two groups as a pair and it is decided by chance (like the flip of a coin).
2. Everyone in the study will be asked to complete a survey in the beginning. We will be available to answer any questions you have while completing your first survey. At 6, 12 and 18 months a survey will be mailed to you and your caregiver, to complete and return to us in a self-addressed stamped envelope. If we do not receive the completed survey in the mail, we will call you.
3. In addition:
   a. If your pair is randomly assigned to group one you will receive an information packet from your local Aging and Disability Resource Center with information and resources in your community.
   b. If your pair is randomly assigned to group two, you will be given access to the Elder Tree website for 12 months and the research team will look at how you use the site. Elder Tree is designed to support older adults and caregivers so that older adults can maintain their independence in their home. If you do not have the technology to access Elder Tree we will provide it to you free of charge. We will also bring it to your home and train you on how to use it. There is no cost to you to be involved and if anything happens to the equipment we loan you, you are not responsible if it is stolen or broken. At the end of 12 months the research team will schedule a time to come pick up the device and get your feedback.

How will we protect your confidentiality?

We will do all we can to keep your records confidential. This is very important to us. Only the study researchers organizing and recruiting will have your name. A number will be used on the surveys instead of your name so that you cannot be identified. Any records that have your name on it will be stored in a locked file cabinet at the researchers' office at UW-Madison. Participants assigned access to the Elder Tree website will be required to choose an anonymous codename and password.

Only if the researchers witness or suspect abuse or neglect in the home they may need to discuss the situation with others on the team to explore options to help you.
I _____________________________________(print) agree to allow the University of Wisconsin Madison CHESS Research team into my house to complete the intake interviews related to the Healthy Aging study that I am participating in.

Will there be any Costs?

There is no financial cost to you to participate in this study. The only cost to you is your time spent using the technology and completing surveys beginning today and at 6, 12 and 18 months.

IRB Approval Date: 2/14/2014
Date IRB Approval Expires: 2/13/2015
FWA00005399 ED/SBS IRB
University of Wisconsin – Madison

What are the Benefits?
There is no direct benefit to you for participating in this study. However, the results of this study may benefit older adults by helping researchers discover new ways to support them and their families.

Are there any Risks?
- There will be a slight risk for a breach of confidentiality, as we will be collecting written consent.
- Elder Tree could give you wrong information. However, a panel of experts reviews it.
- You could get wrong information from the Internet and/or discussion group. However, we will provide you with simple tips to help you figure out whether you can trust the information.
- It is possible you could get upset from a posting in the online discussion group. The study team will routinely monitor discussion groups and appropriate action will be taken if a questionable post has been made online and/or if posts may put an individual at risk.
- You may feel a sense of loss when we collect any devices loaned to you for the study.

Will I be Compensated?
As the older adult in this study you have the primary role in using the Elder Tree site so you will be paid $10 for each survey you complete and return. There will be a total of four surveys.

What if I Decide Not to Take Part?
You are free to withdraw from this study at any time. Your decision to withdraw will have no effect on any other service or program provided to you by the UW or your local ADRC.

Before you sign this form, please take as much time as you need to think this over and to ask questions.

Authorization: I ___________________________________have read this consent form, asked questions, and been given answers. I agree to participate in this research study.

My signature also indicates that I have received a copy of this consent form.

___________________________________ ______________________
Signature Date

___________________________________ ______________________
Signature Date
Signature of Principal Investigator or Person Obtaining Consent

FOR ADDITIONAL INFORMATION, please contact:
Dr. David H. Gustafson or Alice Pulvermacher, M.S.
Center for Health Enhancement Systems Studies (CHESS)
University of Wisconsin—Madison
1513 University Avenue
Madison, WI 53706
PHONE (608) 263-4882 or (608) 262-8448
Website: http://www.chess.wisc.edu

You may also contact the Social and Behavioral Science IRB Office at 608-263-2320.

IRB Approval Date: 2/14/2014
Date IRB Approval Expires: 2/13/2015
FWA00005399 ED/SBS IRB
University of Wisconsin – Madison
